# Small-Pox

**Published:** 1894-04-07

**Authors:** 


### Medical Progress.

### SMALL-POX.

Dr. Porter[1] raises some important points. (1) At what age is compulsory vaccination most beneficial? (2) Is the effect of vaccination in the second decade of life permanent, or is it annulled by severe illnesses, typhoid, &c.? (3) Will vaccination run its course if performed during an attack of small-pox? He inclines to prefer the third quinquennium, which obviates also some sentimental objections to vaccination, but thinks severe illnesses may destroy immunity. He shows that vaccinia and variola may run their course together.

Purpuric symptoms after vaccination[2] are mentioned in seven cases with insomnia and fever, and in some there were hæmorrhages from the mucous membranes. Vaccination in hot climates or after a long interval may be effected by preserving the scab from a vesicle,[3] moistening and rubbing it on a scarified surface. Auché[4] claims to have aborted one case of small-pox by injections of serum from convalescents. In another he believes they were used too late.

Guy and Charrin[5] show that immunity means that certain microbes cannot grow in the body, not that the tissues are protected from their toxines. Thus by injection of toxine the Snellen-Schiff reflex is retarded as much in vaccinated as in other rabbits. Lindholm, Svendson, and Finsen[6] show that if the ultra violet rays are excluded from small-pox hospitals by red glass no suppuration occurs, and the vesicles dry up without pitting.

The evidence as to ærial conveyance of small-pox was minutely reviewed by Dr. McVail[7] at the Epidemiological Society. He believes on the whole that the infection may be conveyed several hundred yards under favourable conditions, affecting the contagium, the atmosphere, and the population, but it was pointed out in the discussion that it was almost impossible to eliminate the factor of personal agencies in carrying the disease.

The Salford epidemic, 1892-3,[8] showed a mortality five times as great in unvaccinated as in vaccinated. No deaths occurred in vaccinated persons under twenty, but the immunity appeared to wear out with advancing years.

The *Lancet*[9] points out that there is no difference in efficacy between calf and human lymph, and greater safety in the latter unless the source is well-known. Some of the earlier evidence before the Commission has just been published. Mr. Stansfield thought that the abolition of cumulative penalties was the less of two evils, and that the triennial inspection of public vaccinators is hardly satisfactory.

### Scarlet Fever.

The pathology has been reviewed by Dr. W. Dawson[10] in an interesting paper. He lays down that (1) tonsillitis is invariably present in ordinary scarlet fever. It is the first sign; it recurs in relapses, and its severity varies with that of the other symptoms. (2) It is absent just where infection enters by an unusual channel, as in puerperal and surgical cases. (3) People who have normal tonsils have not had scarlet fever, as an attack causes permanent destruction and scarring (4) Susceptibility varies with the growth and atrophy of the tonsils. Thus before six months and after thirty years, when the tonsils may be considered as non-existent, there is almost complete immunity. (5) The disease is therefore primarily a local affection of the tonsil, and the general symptoms depend on absorption of toxins produced there. Berge,[11] of Paris, adopts a similar view, and considers the disease due to the toxins produced by streptoccus pyogenes flourishing in a virulent form in the crypts of the tonsil.

The infection of a child in utero is recorded by Ballantyne and Milligan,[12] who consider that this only happens in some cases of maternal disease. If this has happened and the child is born forthwith, it appears to be in exactly the same stage of the illness as the mother, and presents the same clinical features. The prognosis is grave, but not hopeless. Ricochon[13] narrates a case of scarlatina from which other persons contracted in turn erysipelas, suppurative adenitis, puerperal scarlatina, lymphangitis. Is, therefore, the poison the same in all? A case of recrudescene is reported from the City Fever Hospital.[14] The first attack was on October 16th, the second commenced on December 9th. There were typical symptoms with tonsillitis, &c., in both.

The extractives of the spleen[15] in fatal scarlatina are found to contain a proteid which, when introduced into the circulation, is excreted by the kidneys, and tends to produce in them a parenchymatous inflammation.

An outbreak of 28 cases in Glasgow, December, 1893,[16] was traced to sore throats in the milkmen, which turned out to be of the scarlatina character. Later on Dr. W. A. Jamieson[17] praises a spray of peroxide of hydrogen of ten vol. strength for the throat from three to twelve times daily, with thorough baths and inunctions to obviate infection.

*Nephritis.*—To prevent nephritis, Flint[18] insists on a milk diet, whenever it can be taken well, for at least a month after the fever, with gentle aperients and diaphoretics, and protection from exposure. If milk alone cannot be taken, two meals at least should be composed of it; at any rate, plenty of warm drinks and diuretics must be continued for at least a month. Under this plan no severe case of nephritis occurred in sixty patients.

*Antipyretics.*—T. H. Carslaw[19] has examined the value of antipyretic treatment in severe cases (*a*) of anginous, (*b*) of a neural or collapsing type. He concludes that diaphoretics may be useful in less severe cases, but they, especially pilocarpine, may be dangerous in (*b*). This is even more true of phenacetin and similar drugs. Tepid spongings give much relief in simple and anginous cases; mustard spongings are particularly useful in early stages of neural attacks. The cold pack repeated is the best treatment for hyperpyrexia in these cases, and cold to the head is sometimes helpful, but care is needed in all cold applications. He details cases where temperatures of 106, 107, and 108 were noted.

The frequent occurrence of diphtheria in scarlatina cases at the London infectious hospitals, amounting to 199 in 1892, with a mortality of 45 per cent., is a serious matter, the cause of which is not always clear. 295 cases notified as scarlatina were found to be instances of measles, epidemic rose rash, tonsillitis, and other diseases, and 6·3 per cent. of small-pox cases were also found to be errors.

Drs. Washbourn and Goodall[20] show from bacterial investigation that membranous inflammation of the throat in acute stages of scarlatina is usually not diphtheritic, but in the convalescent stage it is. The scarlatinal membrane does not spread to the larynx or recur. Diphtheritic rashes are darker, and almost always limited to the trunk.

*Influenza* is declared by Clemow[21] in a paper full of historic details to be always endemic in Russia. The great epidemic commenced in Western Siberia, September, 1889, and radiated east and west, north and south, along lines of travel, not faster than would be the case if spread by contagion alone.

In December, 1893, the last epidemic arose simultaneously in America and Europe, but sporadic cases were probably to be found previously everywhere. Pfeiffer's[22] bacillus was detected in the sputa by Neisser, Boschardt, and Huber[23] in a large number of cases, but not in the blood. It served to distinguish the disease from typhoid. Cultivations can be made on agar mixed with hæmatogen or blood agar.

Klein[24] has shown that the bacillus requires an alkaline, or at least neutral medium for growth, and is more sensitive to eucalyptus, izal, &c., than to carbolic or sanitas.

Salipyrin, 7 to 15 grains every night on an empty stomach, is recommended by Von Mosengeil[25]; bichloride of mercury by Thayer[26]; pot. bicarb. gr. xxx. tertiis horis exlacte, for two or three doses, then T.D.S., by Crevar[27]; benzole in mucilage ♏v. binis, by W. Robertson[28]; boraline and various inhalations are praised as preventatives.

[1] B. M. J., Jan. 27, 1894. [2] Amer. J. Med. Sci., Jan., 1894. [3] Med. Rec , Dec. 9, 1893. [4] Amer. J. Med. Sci., Nov., 1893. [5] Med. Rec., Jan. 20. 1894. [6] Med. Rec., Jan. 27, 1894. [7] Lancet, Feb. 3, 17, 1894. [8] Pract., Feb., 1894. [9] Lancet, Feb. 3, 1894. [10] Med. Chron., Jan., 1894. [11] Med. Rec., Jan. 27, 1894. [12] Edin. Med. J., July, and Amer. J. Med. Sci., Dec., 1893. [13] Med. Rec., Feb. 3. 1894. [14] B. M. J., Feb. 3, 1894. [15] Pract., Jan., 1894. [16] Glasgow Med. J., Feb., 1894. [17] Pract , Dec., 1893. [18] N. Y. Med. J., Jan. 6, 1894. [19] Glasgow Med. J., Jan. and Feb., 1894. [20] Lancet, Feb. 10, 1894. [21] Lancet, Jan. 20, Feb. 10, 1894. [22] B. M. J., Feb. 10, 17, 1894. [23] B. M. J., Feb. 10, 17, 1894. [24] B. M. J., Feb. 10, 1894. [25] B. M. J., Dec. 23, 1894. [26] Med. Rec., Feb. 17, 1894. [27] Lancet, Feb. 3, 1894. [28] B. M. J., Dec. 30, 1893.

